# Analysis of plasma metabolomes from 11 309 subjects in five population-based cohorts

**DOI:** 10.1038/s41598-024-59388-7

**Published:** 2024-04-18

**Authors:** Nilanjana Ghosh, Carl Lejonberg, Tomasz Czuba, Koen Dekkers, Richard Robinson, Johan Ärnlöv, Olle Melander, Maya Landenhed Smith, Anne M. Evans, Olof Gidlöf, Robert E. Gerszten, Lars Lind, Gunnar Engström, Tove Fall, J. Gustav Smith

**Affiliations:** 1 The Wallenberg Laboratory/Department of Molecular and Clinical Medicine, Institute of Medicine, Gothenburg University and the Department of Cardiology, Sahlgrenska University Hospital, SE-413 45 Gothenburg, Sweden; 2 Department of Cardiology, Clinical Sciences, Lund University, Lund, Sweden; 3 Molecular Epidemiology, Department of Medical Sciences, Uppsala University, Uppsala, Sweden; 4 Metabolon Inc, Morrisville, NC 27560 USA; 5 Division of Family Medicine and Primary Care, Department of Neurobiology, Care Sciences and Society, Karolinska Institutet, Huddinge, Sweden; 6 Department of Internal Medicine, Clinical Sciences, Lund University, Malmö, Sweden; 7 Department of Molecular and Clinical Medicine, Institute of Medicine, Gothenburg University and the Department of Cardiothoracic Surgery, Sahlgrenska University Hospital, Gothenburg, Sweden; 8 Division of Cardiovascular Medicine, Beth Israel Deaconess Medical Center and Harvard Medical School, Boston, MA USA; 9 Department of Medical Sciences, Uppsala University, Uppsala, Sweden; 10 Cardiovascular Epidemiology, Clinical Sciences, Lund University, Malmö, Sweden; 11 Department of Heart Failure and Valvular Disease, Skåne University Hospital, Lund, Sweden; 12 Wallenberg Center for Molecular Medicine and Lund University Diabetes Center, Lund University, Lund, Sweden

**Keywords:** Metabolomics, Molecular medicine

## Abstract

Plasma metabolomics holds potential for precision medicine, but limited information is available to compare the performance of such methods across multiple cohorts. We compared plasma metabolite profiles after an overnight fast in 11,309 participants of five population-based Swedish cohorts (50–80 years, 52% women). Metabolite profiles were uniformly generated at a core laboratory (Metabolon Inc.) with untargeted liquid chromatography mass spectrometry and a comprehensive reference library. Analysis of a second sample obtained one year later was conducted in a subset. Of 1629 detected metabolites, 1074 (66%) were detected in all cohorts while only 10% were unique to one cohort, most of which were xenobiotics or uncharacterized. The major classes were lipids (28%), xenobiotics (22%), amino acids (14%), and uncharacterized (19%). The most abundant plasma metabolome components were the major dietary fatty acids and amino acids, glucose, lactate and creatinine. Most metabolites displayed a log-normal distribution. Temporal variability was generally similar to clinical chemistry analytes but more pronounced for xenobiotics. Extensive metabolite-metabolite correlations were observed but mainly restricted to within each class. Metabolites were broadly associated with clinical factors, particularly body mass index, sex and renal function. Collectively, our findings inform the conduct and interpretation of metabolite association and precision medicine studies.

## Introduction

The collection of small molecules involved in metabolic reactions throughout the human body, influenced by dietary intakes, medications and other environmental exposures, is referred to as the metabolome. Profiling of the metabolome represents a potentially powerful tool to monitor homeostatic processes and disease states that may serve to guide diagnosis and therapy (precision medicine) for many diseases^1^. Given the infeasibility of obtaining samples from most human tissues, plasma is often used as a singular representation of the overall metabolic state of human tissues. Recent studies have described plasma metabolomic profiles associated with cardiovascular disease, cancer and metabolic disease^2,3^. However, limited information is available on the distributional properties of the plasma metabolome in the general population. In addition, several key analytical issues have been described for metabolomic data, including temporal variability, substantial metabolite collinearity, and association with many clinical factors that may confound associations, but the extent of these issues in plasma samples across multiple population-based studies have not been well described^4^.

Mass spectrometry is the gold standard method for comprehensive analysis of the metabolome, with high sensitivity, specificity and capacity for unbiased and high-throughput discovery and quantitative assessment of the components of the metabolome^5^. Several mass spectrometric platforms are available commercially, which differ in metabolite coverage due to differences in instrument setup, sample preprocessing, and reference library used for metabolite identification. Previous work from the Consortium of Metabolomics Studies (COMETS), seeking to combine metabolomic data from multiple cohorts, described the challenges of combining cohorts generated on different platforms^6^.

Here, we therefore describe the profiling of 5 population-based cohorts totaling 11,309 subjects using an untargeted mass spectrometry platform with high metabolite coverage^6^. Cohorts display differences in demographic characteristics, sample collection timepoint, and storage time. We explore the detected plasma metabolome components across these cohorts after an overnight fast, their distribution across cohorts, as well as the main analytical issues that have been described for metabolomic data. Our findings may inform the conduct of studies comparing plasma metabolomic profiles across different conditions and in the combined analysis of multiple cohorts.

## Results

### The human fasting plasma metabolome across cohorts

The distribution of demographic and clinical factors in the five cohorts is shown in Table 1. All cohorts included equal proportions of men and women, and mainly included middle-aged subjects with age range 50–80 years, while two cohorts were age-specific: PIVUS in which all participants were 80 years old and POEM in which all were 50 years old.

**Table 1:** Baseline characteristics of cohorts.

	MDCS	PIVUS	POEM	SCAPIS-M	SCAPIS-U
Site of collection	Malmö, Sweden	Uppsala, Sweden	Uppsala, Sweden	Malmö, Sweden	Uppsala, Sweden
Years	1991–1996	2011–2014	2010–2016	2014–2018	2015–2018
Number (n)	1,083	605	502	4,133	4,986
Age (mean ± SD)	58.19 ± 5.99	80	50	57.49 ± 4.29	57.63 ± 4.39
Female sex (n, %)	529 (52)	299 (49)	249 (50)	3322(53)	2,585 (51)
BMI (mean ± SD)	26.01 ± 4.08	26.91 ± 4.52	26.47 ± 4.29	27.31 ± 4.66	27.04 ± 4.38
Current smoking n (%)	30 (2)	18 (3)	49 (10)	1,087 (17)	471 (9)
eGFR (mean ± SD)	76.17 ± 13.57	62.36 ± 14.61	96.32 ± 10.95	84.86 ± 12.32	87.27 ± 11.45
CRP (mean ± SD)	5.7 ± 3.6	3.15 ± 6.89	1.97 ± 2.86	2.41 ± 4.38	2.18 ± 4.29
Hemoglobin (mean ± SD)	142.26 ± 17.38	136.35 ± 11.29	137.55 ± 11.60	142.67 ± 12.17	141.76 ± 11.75

Characteristics of participants in each of the five study cohorts: the Malmö Diet and Cancer Study (MDCS), the Prospective Investigation of the Vasculature in Uppsala Seniors (PIVUS), the Prospective investigation of Obesity, Energy and Metabolism (POEM), and the Swedish Cardiopulmonary Imaging Study cohorts from Malmö and Uppsala (SCAPIS-M and SCAPIS-U). Continuous variables are presented as mean and standard deviation, and categorical variables as count and percentage. BMI, body mass index. CRP, C-reactive protein. eGFR, estimated glomerular filtration rate.

**Figure 1:**
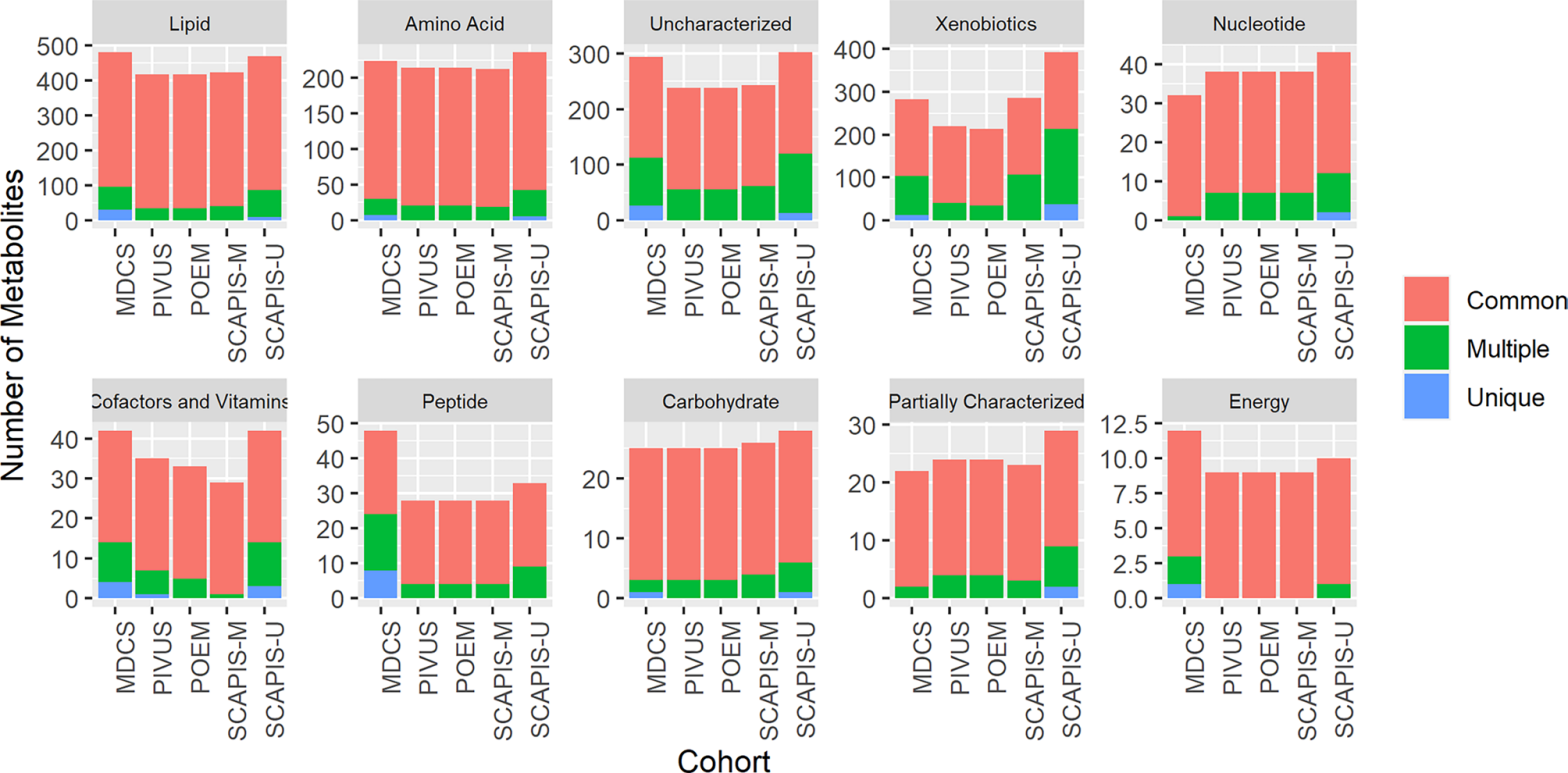
Number of plasma metabolites detected in each cohort. The proportion of metabolites commonly detected in all cohorts, in multiple cohorts, or that were unique to one cohort are indicated by coloring.

In total, we detected 1629 unique metabolites in at least one cohort (Supplementary Table 1), of which most (1074, 66%) were detected in all five cohorts (Fig. 1), while 165 (10%) were unique to only one cohort. Of the 1074 metabolites detected in all five cohorts, 867 (81%) were found in > 50% in all cohorts. The largest metabolite classes consistently detected in all cohorts were lipids (464 metabolites) and amino acids (234 metabolites), while substantial proportions of metabolites were only classified as xenobiotics (357, 22%) or were uncharacterized (306, 19%).

**Figure 2:**
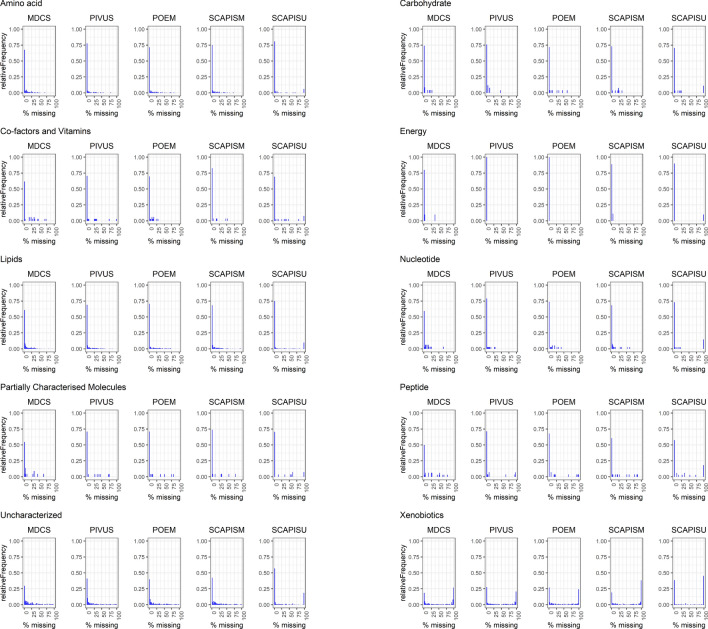
Distribution of metabolite missingness across cohorts. Proportion of metabolites detected in different proportions of each cohort, where 0% missingness indicates detection in all cohort participants.

**Figure 3:**
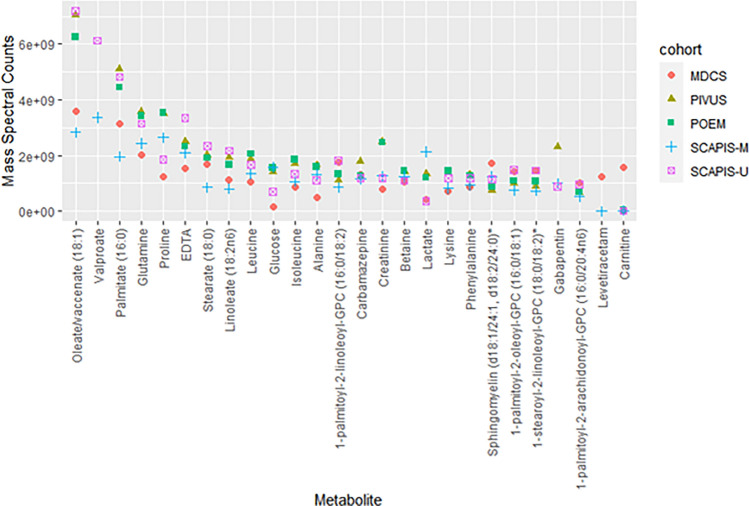
High signal abundance metabolites. The 27 metabolites with mass spectral count > 1 billion units in at least one cohort, with median count from each cohort presented, ordered by median count across all cohorts.

The proportion of metabolites with missing data across different classes is shown for each cohort in Fig. 2. The percent missingness trend was similar across all cohorts, and was generally low except (< 10%) for uncharacterized and xenobiotic metabolite classes. Of the 10% of metabolites only observed in one cohort most (54%) were xenobiotics or uncharacterized (Supplementary Table 1). Similarly, of metabolites with > 80% missingness in any cohort, 76% were xenobiotics or uncharacterized. A smaller set of metabolites consistently ranked as having the higest spectral count (here used as surrogate for metabolite concentration) across all cohorts (Fig. 2), including the major dietary fatty acids (oleate, palmitate), several particularly important amino acids (glutamine, its derivative proline, branched-chain amino acids leucine and isoleucine), and creatinine all of which were present at more than 500-fold higher mass spectral count than the average metabolite and 20 000-fold higher than the metabolites with the lowest count. The carbohydrates glucose and lactate were also consistently amongst metabolites with the highest count, although with lower count in the MDCS for which samples have a 20 years longer storage time than the other cohorts and glucose is well known to decrease with storage time (Fig. 3)^7^.

**Figure 4:**
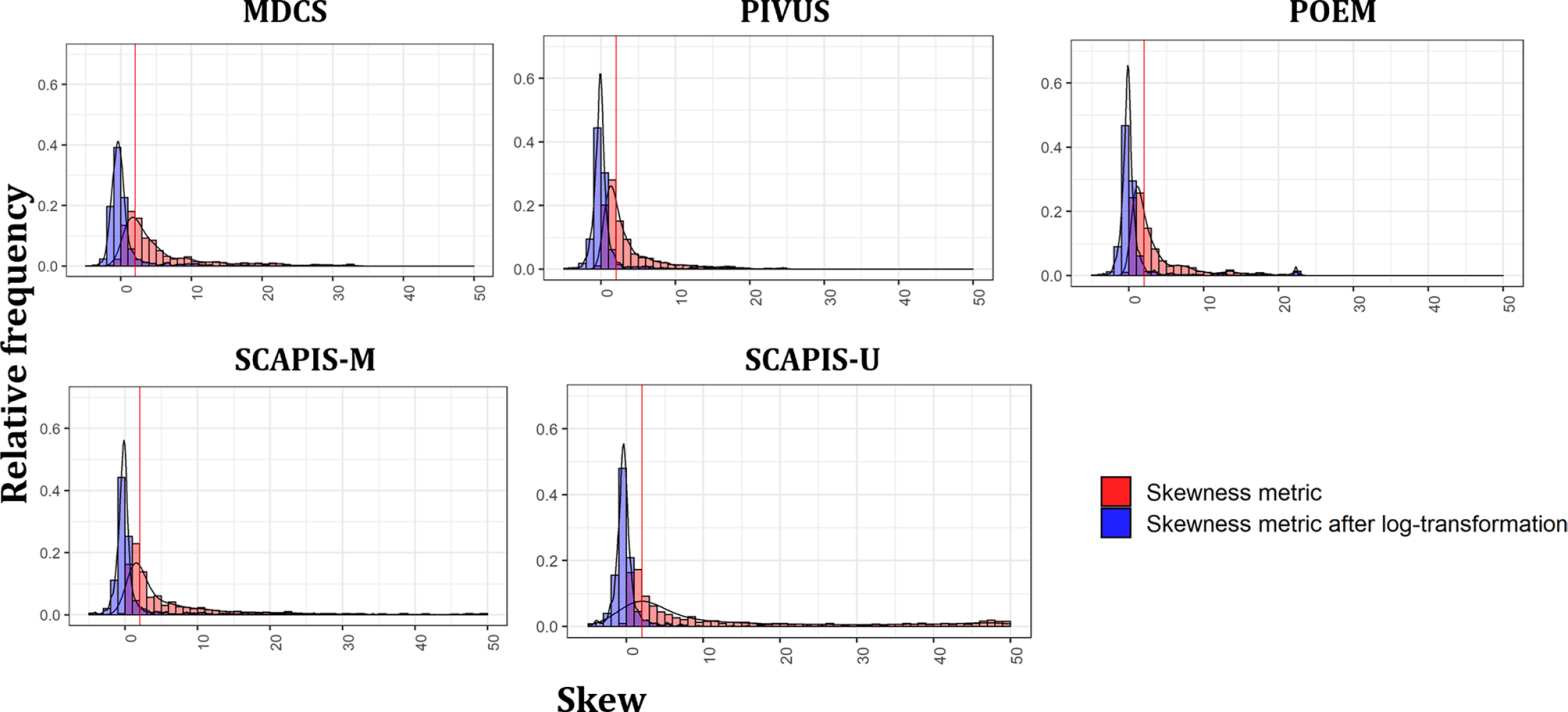
Presence of skewed distribution in plasma metabolites before and after log-transformation. Proportion of metabolites with different levels of positive and negative skewness in each cohort, where 0 indicates absences of skewness.

We next explored metabolite distributions across cohorts and observed widespread skewness of metabolites in all cohorts (Fig. 4), with a skewness measure > 2 (positive skewness) observed in 37% (SCAPIS-U) to 54% (MDCS) of the detected metabolites. No metabolites displayed evidence of negative skewness. Log-transformation largely removed the positive skewness, with a measure > 2 remaining in between 6% (POEM) to 12% (SCAPIS-M). Skewness was similarly high across different metabolite classes (Supplementary Table 1).

**Figure 5:**
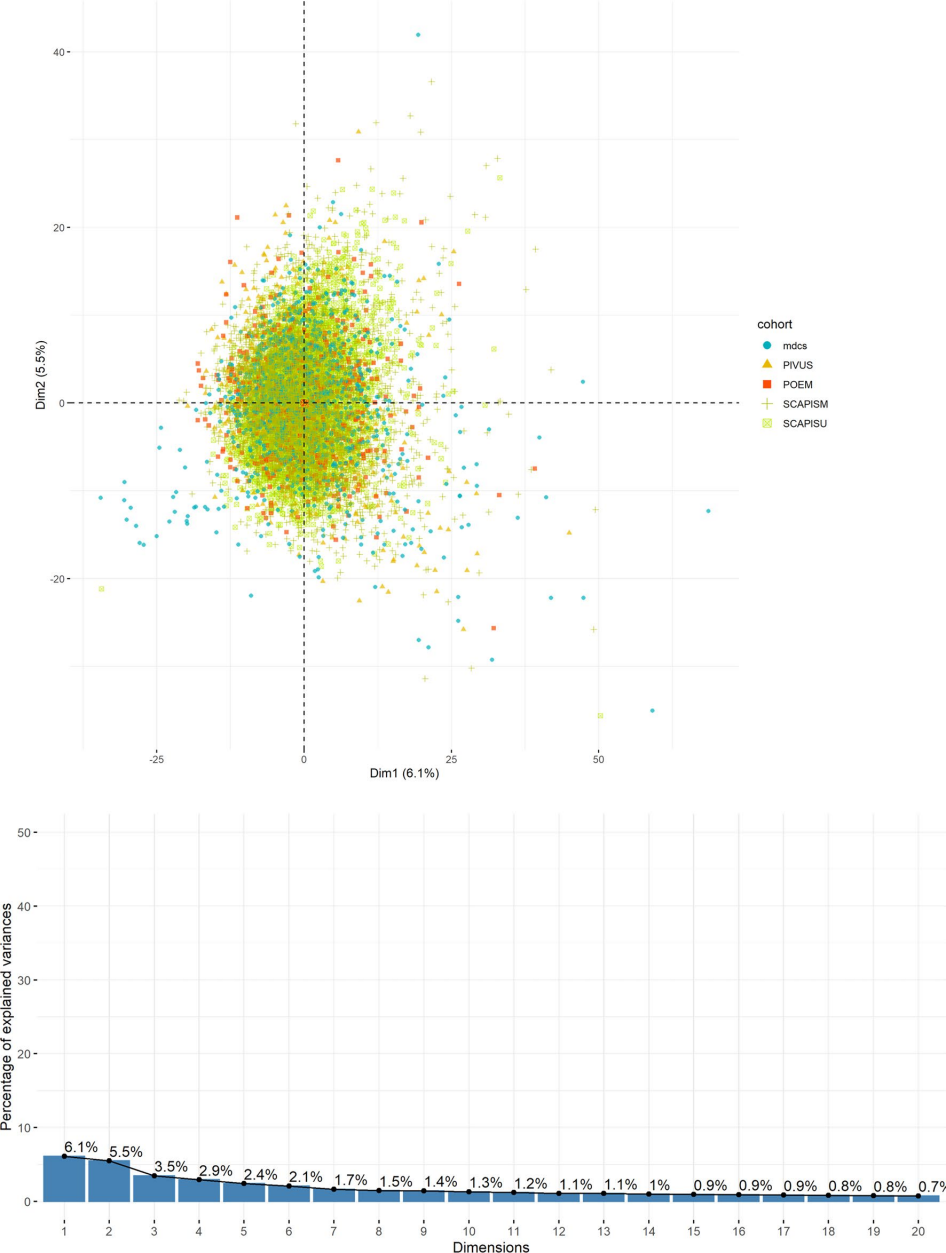
Principal components of the plasma metabolome across cohorts. The first two components from principal component analysis incorporating metabolites with < 50% missingness from all cohorts, and the proportion of variance explained by each of the 20 first components.

Principal component analysis was performed on a core set of 867 metabolites which were commonly present in all five cohorts with less than 50% missingness. (Fig. 5) No evidence of systematic differences in metabolite profiles between cohorts was observed, and principal components explained only a small proportion of variability in the metabolome (≤ 6.1%). Interestingly, principal component 1 (6.1%) associated with many metabolites but most strongly with DMTPA (r^2^ = 0.79) and urate (r^2^ = 0.71) (Supplementary Table 2), both of which are known to relate strongly to renal function.

### Metabolite variability over one year

**Figure 6:**
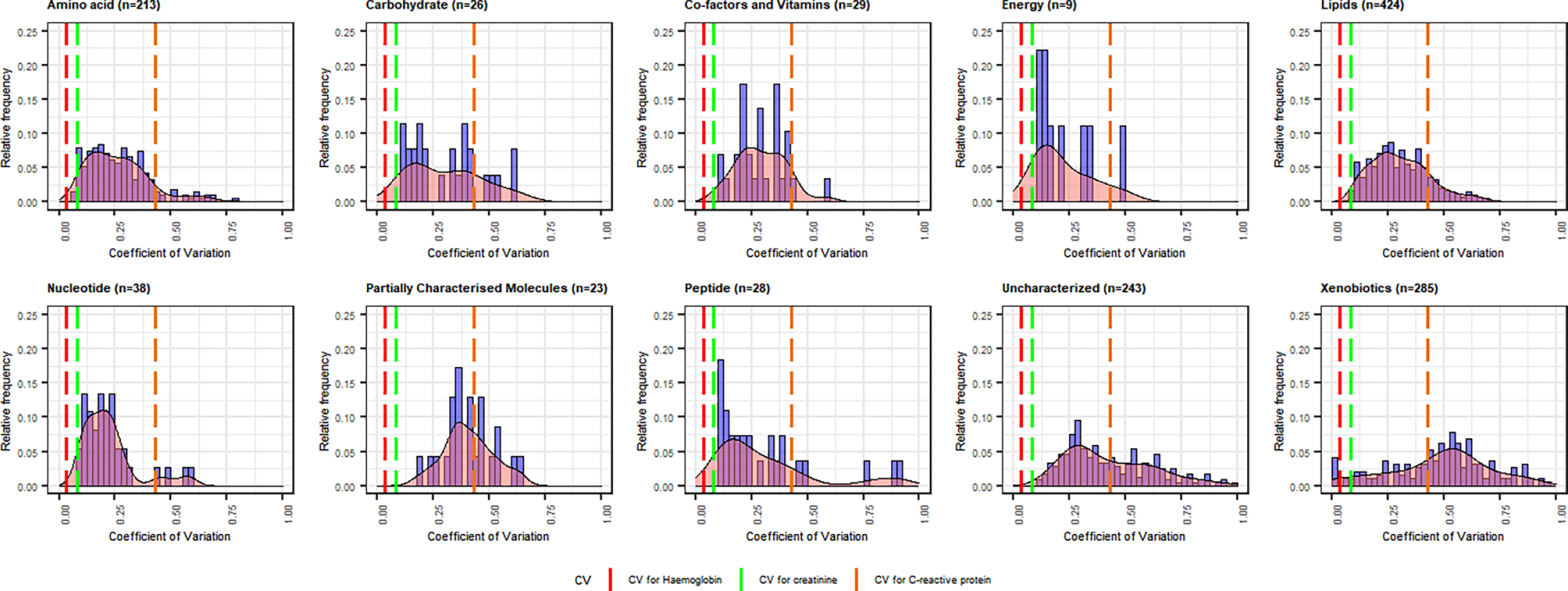
Plasma metabolite variability over one year. Distribution of coefficients of variation for individual metabolites, with variation over time (based on individual mass spectral counts) in proportion to the average, where 0 indicates that a metabolite does not vary over time while 1 indicates that variability over time is as large as the mean mass spectral count. For comparison, the coefficients were 0.03 for hemoglobin (ref), 0.08 for creatinine (green), and 0.42 for C-reactive protein (orange).

A second sample was obtained from the same subjects after 1 year in SCAPIS-M, in 147 randomly selected subjects to explore variability over time. Variability over time was in the same range as that observed for common routine clinical assays in SCAPIS-M for hemoglobin (0.03), creatinine (0.08) and CRP (0.42), with a median coefficient of variation ranging from 0.20 in nucleotides, 0.23 in peptides, 0.25 in amino acids, 0.29 in co-factors and vitamins, 0.29 in lipids, 0.31 in carbohydrates, but higher for xenobiotics (0.53) (Fig. 6). Temporal variation for each metabolite is presented along with between-subject variability at baseline in Supplementary Table 1. Importantly, between-subject variability was larger than within-subject variability for 99% of metabolites (median 94% higher).

### Pairwise metabolite-metabolite correlations

**Figure 7:**
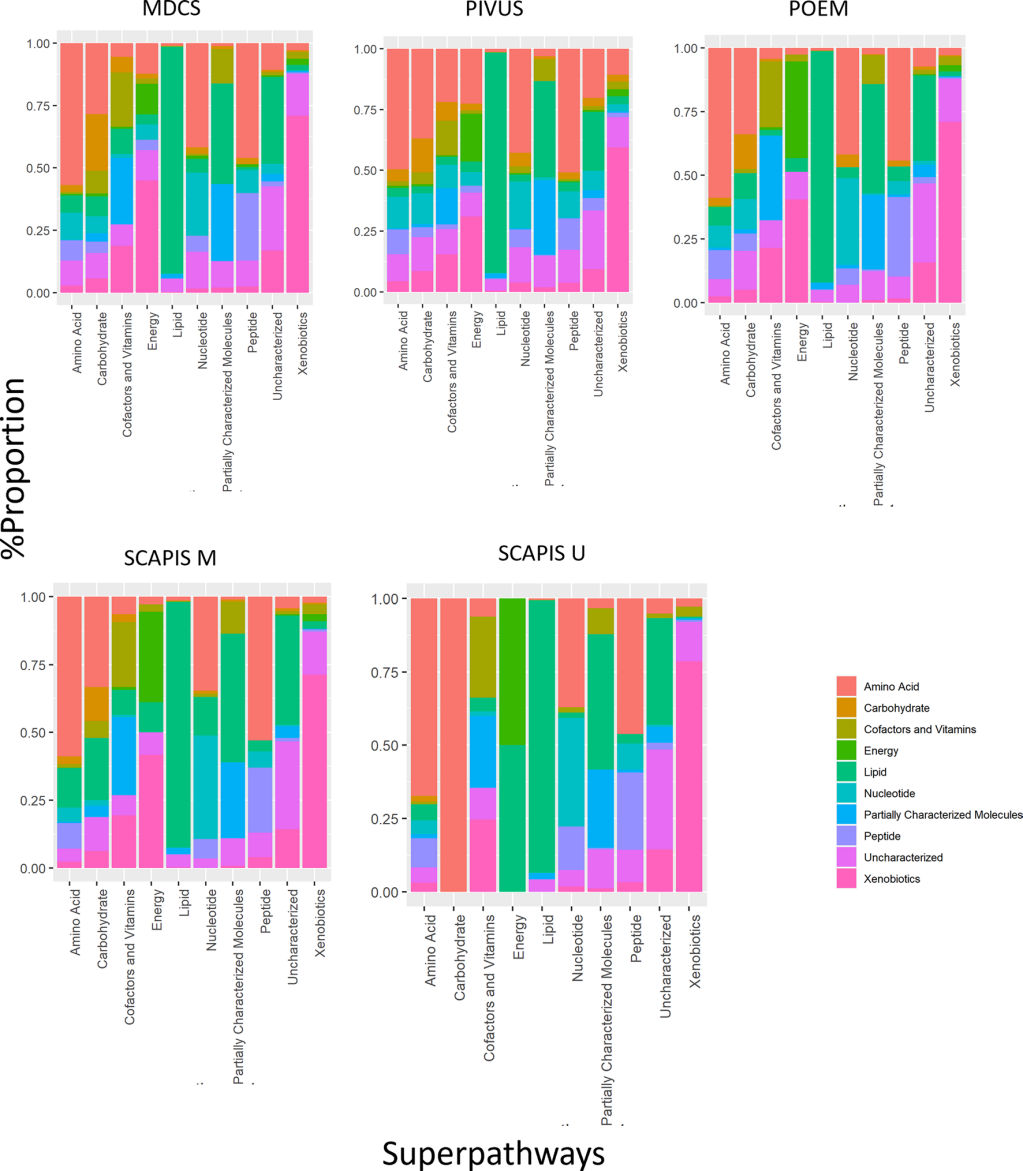
Pairwise metabolite-metabolite correlations. Proportion of high-level correlations for metabolites in each biochemical class based on class of the corresponding highly correlated (r > 0.5) metabolites.

We next examined the patterns of correlation between pairs of metabolites within each of the five cohorts, based on the 867 metabolites present in > 50% of participants from each cohort. As expected, significant metabolite correlations were pervasive and the number of high-level correlations was proportional to the size of the biochemical class, such that the classes with most metabolites detected (lipids, amino acids, xenobiotics) displayed the largest numbers of correlations. Importantly, most (99%) highly correlated metabolite pairs (r > 0.5) (Supplementary Table 3) were restricted to metabolite pairs within the same biochemical class, with a particularly high number of correlations within the large lipid group (93% of all high correlations), which was consistent across all cohorts. Figure 7 displays the 2213 metabolites with consistently high correlation (r > 0.5) across all cohorts, from a total of up to 575,128 possible correlations.

### Association of metabolites with clinical factors

**Figure 8:**
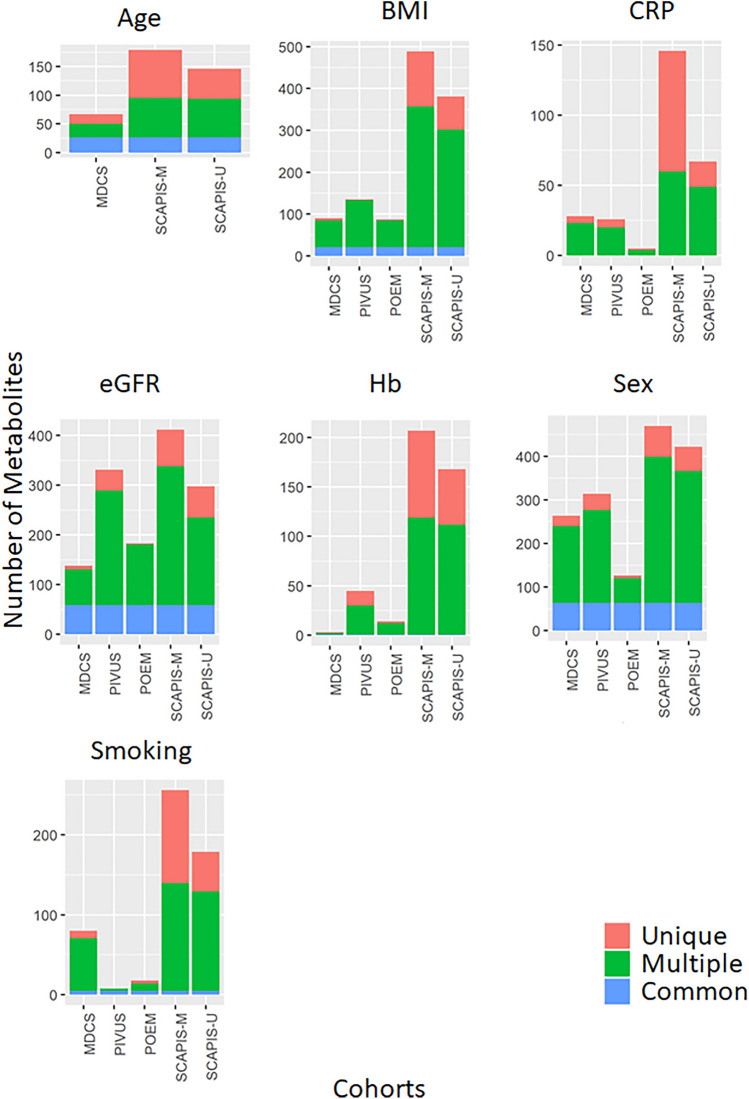
Association of plasma metabolites with clinical factors. Number of plasma metabolites associated with each of seven important clinical factors in each cohort, at a significance threshold Bonferroni-corrected for the total number of statistical tests (*p* < 8 × 10^−6^). Colors indicate whether associations were significant across all cohorts (blue), in multiple cohorts (green) or unique to one cohort (red). As expected, the number of significant associations scales with cohort size.

**Figure 9:**
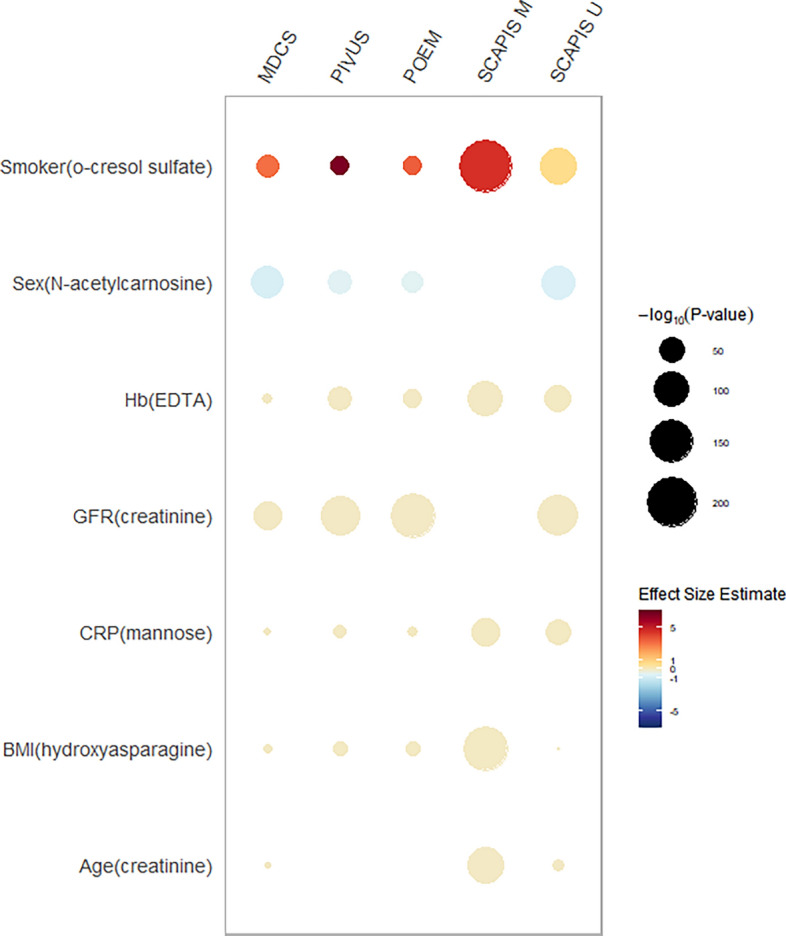
Top associations with clinical parameters across cohorts. Results from each cohort for metabolites for each clinical parameter that displayed the strongest associations in the large SCAPIS-M cohort, adjusted for age and sex. Effect estimates were beta estimates per log-standard deviation of each metabolite from regression models.

We next explored the association with central clinical factors for the 867 metabolites present in > 50% of participants from all cohorts. We observed pervasive association of metabolites with clinical factors at a Bonferroni-adjusted significance threshold of 8 × 10^−6^, with nearly half of all metabolites associated with BMI, sex and renal function, but also with 10–20% of metabolites for age, smoking, CRP, and hemoglobin (Fig. 8). As expected, the proportion of significant associations was highest in the two largest cohorts, SCAPIS-M and SCAPIS-U. Most significant associations were detected in at least these two cohorts (Fig. 8), and effect estimates were typically concordant across all cohorts as shown for the strongest associations with each trait in SCAPIS-M in Fig. 9 and for all metabolites in each cohort in Supplementary table 4. For smoking, the strongest association was observed the phenylsulfate o-cresol sulfate, which together with cotinine metabolites (which were also strongly associated with smoking) has been consistently associated with smoking also in previous studies^8^.

For age and renal function, the strongest association was with creatinine, a metabolite of muscle creatine which is routinely used in the clinic to estimate renal function and well known to correlate closely with age-related declines in muscle mass^9^. For hemoglobin, these strongest association was with ethylenediaminetetraacetic acid (EDTA), an exogenous synthetic compound used in blood tubes to prevent coagulation. The finding that hemoglobin concentration, reflecting less tube coagulation, relates to EDTA levels is thus to be expected. The strongest association for BMI, (hydroxyasparagine, a derivative of the amino acid asparagine) has also been reported previously^10^.

## Discussion

Our analysis of untargeted fasting plasma metabolomes of > 11,000 human subjects from five population-based cohort studies found striking similarities across endogenous metabolomes. The metabolites present in plasma at highest quantity were consistently found to be the major dietary fatty acids followed by particularly important amino acids. This is not surprising, considering estimations that in addition to water, which makes up almost two thirds of the human lean body mass, most of the remaining mass represents lipids and amino acids (each 15%) for which large depots are present in the human body^11^. Depots for carbohydrates (glycogen) and trace substances are substantially smaller, only about 5%. The fatty acids consistently detected with the highest signal in fasting plasma, oleate and palmitate, are known to be the most widely distributed fatty acids in nature and in human foods^12^. Stearate and linoleate were also consistently amongst the metabolites with highest signal. The most strongly detected amino acids were glutamine, an important energy source derived from main dietary amino acid glutamate in skeletal muscle^13^, its derivative proline—the major component of collagen^14^, and the essential dietary branched-chain amino acids leucine and isoleucine which are important for muscle metabolism. The energy carriers glucose and lactate and phosphocreatine synthesis byproduct creatinine were also present with robust signal as expected.

Our findings have several implications for the analysis of population-based metabolomics data. A key issue in the analysis of metabolomic data is the level of metabolite missingness^15,16^. Such missingness may have different biological interpretations based on whether the metabolite of interest is not present (as in the case of drugs and exposure to toxic metals) or simply below the threshold of detection (as in the case of intracellular metabolites which only circulate at low levels). In the former case, minimum value interpretation, as often used in metabolomics studies, is not valid while in the second case minimum value interpretation would be expected to contribute to improve statistical power. In addition to these two situations of non-random missingness, missingness in a smaller subset of samples may be due to random data processing errors or systematic non-random errors such as inaccurate peak detection. Such errors are hard to recognize in the analytical stage. However, our findings suggest that marked missingness in the fasting plasma metabolome is largely restricted to xenobiotic metabolites, likely representing absence of these compounds in most subjects, which should therefore be treated separately in many analyses. Indeed, it is widely accepted that 20% missingness or more should be handled separately, referred to as the “80% rule”^17^. A second issue is the distribution of values of metabolites, which impacts parametric assumptions and model specification. Our findings show that although most metabolites consistently display a positively skewed distribution across cohorts, a simple logarithmic transformation results in marked attenuation of such skewness resulting in approximate satisfaction of normality assumptions. The lack of metabolites with negatively skewed distribution may reflect limitations in assay sensitivity for detection of low values combined with the use of minimum value imputation in subjects with missing values. A third issue is the presence of abundant correlation between metabolites, potentially resulting in multicollinearity or confounding. Our findings suggest that although widespread, high-level metabolite-metabolite correlations in the fasting plasma metabolome are mainly restricted to within chemical classes and pathways. A fourth issue is variability over time. We observed similar variability of metabolites to routinely used clinical chemistry analytes within the same individual over one year, in the fasting state, which however was more marked for xenobiotic metabolites again suggestive that these need to be treated separately. Furthermore, within-subject variability over one year was markedly lower than between-subject variability.

Finally, of larger concern, we also observed extensive associations of metabolites with clinical factors, potentially resulting in confounded, false positive associations through omitted variable bias if not controlled for. As expected, with increased sample size the number of significant associations increased, with up to 400–500 metabolites associating with BMI, renal function and sex.

Limitations to the current study include the use of only cohorts representing the Swedish population, with a predominance of middle-aged, mainly healthy participants. Nevertheless, our study represents the largest reported analysis of the plasma untargeted metabolome generated to our knowledge and the cohorts displayed differences in demographic characteristics, sample collection timepoint, fasting state, and storage time. Although we did not explore the impact of fasting times, it seems likely that non-standardized fasting states would provide markedly increased variation in metabolite levels. The majority of human plasma metabolomics studies have used fasting plasma and in-depth studies are needed to clarify how fasting versus fed status, fasting time, dietary patterns, and circadian cycles impact the plasma metabolome^18^. We also advise caution in imputation and analysis of certain xenobiotics/drugs such as Carbamazepine, Gabapentin, and Levetiracetam that ionize very well and therefore give robust signals in compliant study subjects. Minimum value imputation of signal may yield a skewed view of relative abundance since even non-takers are assigned the lowest level seen in someone who is taking the medication.

## Conclusion

The current study provides information on the components of the human plasma metabolome across cohorts and analytical considerations which may serve to guide interpretation of metabolite association studies.

## Material and methods

### Population-based cohorts

Plasma samples obtained from 11,309 unique participants of five population-based cohort studies underwent mass spectrometry-based metabolomic analysis at a core laboratory (Metabolon Inc., Morrisville, NC, USA). In addition, 147 subjects from one cohort (the Malmö cohort of the Swedish Cardiopulmonary Imaging Study [SCAPIS-M]) underwent analysis of a second sample obtained from the same subjects one year after the baseline visit to allow analysis of temporal variability. In all cohorts, plasma was obtained by centrifugation of blood immediately after sampling into EDTA tubes from a peripheral antebrachial vein in all cohorts and remained frozen to -80° C until analysis without previous thawing. Analysis of all cohorts was approved by the Swedish Ethics Review Authority and all participants provided written informed consent.

#### MDCS

The Malmö Diet and Cancer Study (MDCS) is a population-based cohort study which includes 30,447 men (born between 1923 and 1945) and women (born between 1923 and 1950) from the city of Malmö in southern Sweden with the aim to study risk factors for cancer and cardiovascular disease^19^. Participants underwent baseline examinations between 1991 and 1996, during which anthropometric measures were obtained and a questionnaire was filled out^20^. From this cohort 6,103 individuals with a baseline examination between 1991 and 1994 were randomly selected to participate in a study of cardiovascular risk factors, the MDCS Cardiovascular Cohort (MDC-CC), of whom 5543 underwent blood sampling under standardized overnight fasting conditions^21^. Clinical routine assays were used to measure C-reactive protein (CRP, high-sensitive assay from Roche Diagnostics, Basel, Switzerland), hemoglobin content (the cyanmethemoglobin method), and renal function (creatinine with the Jaffé method). Data on current smoking was ascertained as self-reported from the study questionnaire. For the current study, a random subset of samples from the MDC-CC were selected and evaluated for representativity using the gmatch macro in SAS (SAS Institute, Cary, NC, USA)for metabolomic profiling as described previously (n = 1083)^22^.

#### PIVUS

The Prospective Study of the Vasculature in Uppsala Seniors (PIVUS) study was initiated in 2001 with the primary aim to investigate the predictive potential of different measurements of endothelial function and arterial compliance in a random sample of 1000 men and women aged 70 living in the community of Uppsala^23^. The inclusion of subjects in the study was completed in June 2004. In the spring of 2011 the 80-year reinvestigation of the cohort was started. This round was completed by the summer of 2014 and 605 subjects from the original cohort attended, which were included in the present study. For all participants, anthropometric measures, overnight fasting plasma samples and questionnaire information on smoking was obtained. Plasma creatinine, hemoglobin and CRP were measured using routine clinical chemistry multianalyzers.

#### POEM

The population-based Prospective investigation of Obesity, Energy and Metabolism (POEM) study was conducted in inhabitants of Uppsala, Sweden, aged 50 years. Between October 2010 and October 2016, 502 individuals were investigated (50% women). The primary aim was to explore the links between obesity and a wide range of measures of subclinical cardiovascular disease, including whole-body magnetic resonance imaging^24^. For all participants, anthropometric measures, overnight fasting plasma samples and questionnaire information on smoking was obtained. Plasma creatinine, hemoglobin and CRP were measured using routine clinical chemistry multianalyzers.

#### SCAPIS-M and SCAPIS-U

The Swedish Cardiopulmonary Imaging Study (SCAPIS) is a national Swedish population-based cohort study which includes 30,154 randomly selected men and women aged 50–64^25^. The SCAPIS baseline examination was conducted between 2013 and 2018 in six of the largest Swedish cities: Gothenburg, Linköping, Malmö, Stockholm, Umeå and Uppsala. The aims of the SCAPIS study have been described previously^26^ and were to survey contemporary risk factors for cardiovascular and pulmonary disease. Metabolite profiling was undertaken in 4,133 random participants of the Malmö cohort (SCAPIS-M) and 4,986 participants from the Uppsala cohort (SCAPIS-U). Participants underwent assessment of anthropometric measures, filled out a comprehensive questionnaire, and underwent extensive physical examinations including cardiothoracic and abdominal computed tomography angiography. For all participants, anthropometric measures, overnight fasting plasma samples and questionnaire information on smoking was obtained. Plasma creatinine, hemoglobin and CRP were measured using routine clinical chemistry multianalyzers (Roche Cobas and SYSMEX XN-10).

### Metabolite profiling

Plasma samples from all cohorts underwent untargeted metabolomic analysis at a core laboratory using a well-validated mass spectrometry platform (Discovery HD4 platform, Metabolon Inc., Morrisville, NC, USA) between August 2019 and January 2021^27,28^. The platform combines four complementary sample preprocessing protocols and a comprehensive reference library, resulting in quantitative estimates of metabolite abundance (mass spectral counts) for metabolites covering a broad spectrum of chemical classes, including amino acids, carbohydrates, lipids, nucleotides, peptides and vitamins, but also xenobiotic substances such as pharmaceutical and food preservative compounds. A detailed description of the analytical protocol, metabolite identification, and normalization procedures is included in the Supplementary Methods. Samples were randomized across the platform run with quality control samples spaced evenly among the injections. Metabolite abundance was estimated from the area under the curve for annotated peaks in the mass spectrogram (mass spectral counts) and was normalized for batch and run day in each cohort. All analyses were conducted in individual cohorts and not integrated.

### Statistical analysis

First, we compared the metabolites detected across individual cohorts. Detected metabolites and their missingness were plotted across ten chemical classes: the six main classes were amino acids, carbohydrates, lipids, nucleotides, peptides, and cofactors/vitamins. Biochemicals derived intracellularly as intermediates in the citric acid cycle and oxidative phosphorylation (referred to here as ‘Energy’ metabolites). Xenobiotic chemicals as well as partially characterized or uncharacterized molecules were plotted as separate classes. Metabolites were ranked by median count in each cohort according to raw area under the curve counts, and the distribution was further examined based on quartiles across cohorts. The extent of skewness, as indicator of the validity of parametric assumptions, and effect of natural logarithm transformation was explored across metabolites in each cohort. Principal component analysis was performed across all cohorts, with a covariance matrix based on a core set of metabolites commonly present in cohorts with < 50% missingness to explore systematic differences between cohorts in overall metabolite variation. The plasma metabolome variance explained by individual principal components was derived from eigenvalues and visualized by a scree plot.

Second, we examined metabolite variability over time based on the coefficient of variation between the two sampling timepoints for each metabolite and chemical class. Coefficients were compared to the corresponding numbers for routine clinical assays, including hemoglobin, creatinine, and CRP in the SCAPIS-M cohort.

Finally, we explored the pairwise association between metabolites and of metabolites with clinical factors. The potential for collinearity was explored based on pairwise metabolite correlations in each cohort. Mass spectral metabolite counts were natural logarithm transformed and then centered to the mean and scaled to the standard deviation for ease of comparison in these analyses. The impact of major demographic and clinical characteristics were explored in general linear models, including age, sex, body mass index (BMI), renal function as estimated glomerular filtration rate (eGFR), inflammation as C-reactive protein (CRP), hemoglobin content (Hb) and current smoking. Significance thresholds were Bonferroni-adjusted for the number of tests in each analysis. For metabolite-metabolite correlations and association analyses with demographic factors, we used minimum value imputation to improve statistical power and restricted analyses to the 867 metabolites present in > 50% of participants from each cohort. All data management and analysis was conducted using R Studio Version 1.4.1106.

### Ethical approval

This study was performed in line with the principles of the Declaration of Helsinki. The study and work within each of the contributing cohorts was approved by the Swedish Ethical Review Authority. All participants provided written informed consent.

### Supplementary Information

Supplementary Information 1.

Supplementary Information 2.
