# Supplementary material for: Analysis of plasma metabolomes from 11 309 subjects in five population-based cohorts
Source: Sci Rep. 2024 Apr 18;14:8933. doi: 10.1038/s41598-024-59388-7 (PMC11026396; doi:10.1038/s41598-024-59388-7)
Supplement: Supplementary file 1 — Supplementary Information 1. [file 41598_2024_59388_MOESM1_ESM.docx]

**Analysis of plasma metabolomes from 11 309 subjects in five population-based cohorts**

**SUPPLEMENTARY METHODS**

Nilanjana Ghosh, Carl Lejonberg, Tomasz Czuba, Koen Dekkers, Richard Robinson, Johan Ärnlöv, Olle Melander, Maya Landenhed Smith, Anne M. Evans, Olof Gidlöf, Robert E. Gerszten, Lars Lind, Gunnar Engström, Tove Fall, J. Gustav Smith

**Sample preparation**

Samples were prepared using the automated MicroLab STAR system (Hamilton Company, Reno, NV, USA). Several recovery standards were added before the first step in the extraction. Plasma proteins were precipitated with methanol under vigorous shaking for 2 min (Glen Mills GenoGrinder 2000) followed by centrifugation. The resulting extract was divided into five fractions: two for analysis by two separate reverse phase/ultra-performance liquid chromatography (UPLC)-tandem mass spectrometry (MS/MS) methods with positive ion mode electrospray ionization (ESI), one for analysis by reverse phase/UPLC-MS/MS with negative ion mode ESI, one for analysis by hydrophilic interaction liquid chromatography (HILIC)/UPLC-MS/MS with negative ion mode ESI and one sample reserved for backup. Samples were placed briefly on a TurboVap (Zymark) to remove the organic solvent. The sample extracts were stored overnight under nitrogen before preparation for analysis.

Several types of controls were analyzed in concert with the experimental samples: a pool of well-characterized human plasma served as a technical replicate throughout the dataset; extracted water samples served as process blanks; and a mix of quality control standards that were carefully chosen not to interfere with the measurement of endogenous compounds were spiked into every analyzed sample, allowed instrument performance monitoring and aided chromatographic alignment. Samples were randomized across the platform run with quality control samples spaced evenly among the injections.

**Instrument and process variability**

Instrument variability was determined by calculating the median relative standard deviation for the standards that were added to each sample before injection into the mass spectrometers, and was ~5% in all cohorts. Overall process variability as determined by calculating the median relative standard deviation for all endogenous metabolites (noninstrument standards) present in 100% of the pooled matrix samples was ~10% in all cohorts.

**Mass spectrometry protocol**

All methods utilized an Acquity UPLC (Waters Corp., Milford, MA, USA) and a Q-Exactive high-resolution/accurate mass spectrometer (Thermo Fisher Scientific, Waltham, MA, USA) interfaced with a heated electrospray ionization source and Orbitrap mass analyzer operated at 35,000 mass resolution. The sample extract was dried then reconstituted in solvents compatible to each of the four methods. Each reconstitution solvent contained a series of standards at fixed concentrations to ensure injection and chromatographic consistency. One aliquot was analyzed using acidic positive ion conditions, chromatographically optimized for more hydrophilic compounds. In this method, the extract was gradient eluted from a C18 column (Waters UPLC BEH C18 2.1 × 100 mm, 1.7 μm) using water and methanol, containing 0.05% perfluoropentanoic acid (PFPA) and 0.1% formic acid. Another aliquot was also analyzed using acidic positive ion conditions; however, it was chromatographically optimized for more hydrophobic compounds. In this method, the extract was gradient eluted from the same aforementioned C18 column using methanol, acetonitrile, water, 0.05% PFPA and 0.01% formic acid and was operated at an overall higher organic content. Another aliquot was analyzed using basic negative ion optimized conditions using a separate dedicated C18 column. The basic extracts were gradient eluted from the column using methanol and water, however, with 6.5 mM of ammonium bicarbonate at pH 8. The fourth aliquot was analyzed via negative ionization after elution from a HILIC column (Waters UPLC BEH Amide 2.1 × 150 mm, 1.7 μm) using a gradient consisting of water and acetonitrile with 10 mM of ammonium formate, pH 10.8. The MS analysis alternated between MS and data-dependent MSn scans using dynamic exclusion. The scan range varied slightly between methods but covered 70–1,000 m/z. A detailed description of the gradient elution conditions, injection volume, column temperature, MS/MS fragmentation energy and all other chromatography conditions for the LC-MS methods has been described previously by Ford et al,^1^ particularly in the supplementary tables 1 and 2 of that article.

**Metabolite identification, retention index strategy and normalization**

A detailed description of chromatographic alignment, QC practices, and compound identification has been described previously.^2^ Briefly, for the detection and integration of MS peaks, instrument vendor supplied software is sufficient for generating the lists of peaks detected, organized by mass and time within an instrument data file. In this case, in-house software was used to perform the detection and integration of MS peaks. This software used standard industry approaches for MS peak detection. All samples were aligned based on retention time (RT) markers present throughout the chromatogram using a retention index (RI). The retention index of a sample component was defined as a number, obtained by interpolation (usually logarithmic), relating the adjusted retention volume (time) or the retention factor of the sample component to the adjusted retention volumes (times) of two standards eluted before and after the peak of the sample component”. Briefly, standards or retention markers are spiked into every sample analyzed. These standards are isotopically labeled metabolites chosen for their elution behavior, specifically their retention time and stability.

Raw data were extracted, peak-identified and quality control processed using Metabolon hardware and software.^3^ Compounds were identified by comparison to library entries of purified standards or recurrent unknown entities. Metabolon maintains a library based on authenticated standards that contains the retention time/index, mass to charge ratio (m/z) and chromatographic data (including MS/MS spectral data) on all molecules present in the library. Furthermore, biochemical identifications are based on three criteria: retention index within a narrow retention index window of the proposed identification, accurate mass match to the library ±10 parts per million and the MS/MS forward and reverse scores between the experimental data and authentic standards. The MS/MS scores are based on a comparison of the ions present in the experimental spectrum to the ions present in the library spectrum. While there may be similarities between these molecules based on one of these factors, the use of all three data points can be utilized to distinguish and differentiate biochemicals. Identifications were automatically approved if all the above criteria were met and the MS/MS forward and reverse scores were above 80%. Compounds which met the above criteria but had low MS/MS scores, below 35% for both forward and reverse, were automatically rejected. Compounds with intermediate MS/MS forward and reverse scores, 36 to 79, were marked for manual review. An in-house software package checked all ions that were not assigned to any library entry across a set of injections and within a specified chromatographic time window. All identifications and quantifications were subjected to QC to verify the quality of the identification and peak integration. More than 5,300 commercially available purified standard compounds have been acquired for analysis on all platforms to determine their analytical characteristics. Additional mass spectral entries have been created for structurally unnamed biochemicals, which have been identified by virtue of their recurrent nature (both chromatographic and mass spectral). Library matches for each compound were checked for each sample and corrected if necessary. All named compounds fulfill tier 1 or tier 2 (indicated by an asterisk) criteria according to the metabolomics reporting standards outlined by the Metabolomics Standards Initiative.^4^ Peaks were quantified using the area under the curve. For each cohort, run day normalization was performed to correct for variation resulting from instrument inter-day tuning differences. Each compound was corrected in run day blocks and batches by registering the medians to equal one (1.0) and normalizing each data point proportionately (termed block correction). Each batch represents one analytical plate containing 144 samples.

**Cohort analysis timepoints**

Samples from all cohorts were analyzed between August 2019 and January 2021: MDCS between March – May 2020, SCAPIS-U in two batches of which one (n=1990) between August – September 2019 and a second (n=3000) between February – March 2020, while SCAPIS-M was analyzed between April – June 2020. PIVUS and POEM were analyzed between September 2020 – January 2021.

**References**

1. Ford L, Kennedy AD, Goodman KD, Pappan KL, Evans AM, Miller LAD, Wulff JE, Wiggs BR, Lennon JJ, Elsea S, Toal DR. Precision of a Clinical Metabolomics Profiling Platform for Use in the Identification of Inborn Errors of Metabolism. *J Appl Lab Med*. 2020 ;3:342-356.
2. Evans AM, DeHaven CD, Barrett T, Mitchell M, Milgram E. Integrated, nontargeted ultrahigh performance liquid chromatography/electrospray ionization tandem mass spectrometry platform for the identification and relative quantification of the small-molecule complement of biological systems. *Anal Chem*. 2009; 8:6656-67.
3. Dehaven CD, Evans AM, Dai H and Lawton KA. Organization of GC/MS and LC/MS metabolomics data into chemical libraries. *J Cheminform*. 2010;2:9.
4. Sumner LW, Amberg A, Barrett D, Beale MH, Beger R, Daykin CA, Fan TW, Fiehn O, Goodacre R, Griffin JL, Hankemeier T, Hardy N, Harnly J, Higashi R, Kopka J, Lane AN, Lindon JC, Marriott P, Nicholls AW, Reily MD, Thaden JJ and Viant MR. Proposed minimum reporting standards for chemical analysis Chemical Analysis Working Group (CAWG) Metabolomics Standards Initiative (MSI). *Metabolomics*. 2007;3:211-221.
